# Variation of Circulating Brain-Derived Neurotrophic Factor (BDNF) in Depression: Relationships with Inflammatory Indices, Metabolic Status and Patients’ Clinical Features

**DOI:** 10.3390/life13071555

**Published:** 2023-07-13

**Authors:** Valentina Falaschi, Lionella Palego, Donatella Marazziti, Laura Betti, Laura Musetti, Alessandra Maglio, Valerio Dell’Oste, Simona Sagona, Antonio Felicioli, Barbara Carpita, Alberto Brogi, Federico Mucci, Enrico Massimetti, Liliana Dell’Osso, Gino Giannaccini

**Affiliations:** 1 Department of Clinical and Experimental Medicine, Section of Psychiatry, University of Pisa, Via Savi 10, 56126 Pisa, Italy; valentina.falaschi@uslnordovest.toscana.it (V.F.); dmarazzi@psico.med.unipi.it (D.M.); laura.musetti@unipi.it (L.M.); dralessandramaglio@gmail.com (A.M.); valerio.delloste@gmail.com (V.D.); barbara.carpita@unipi.it (B.C.); federico.mucci@med.unipi.it (F.M.); enrico.massimetti@uslnordovest.toscana.it (E.M.); liliana.dellosso@unipi.it (L.D.); 2 Department of Pharmacy, University of Pisa, Via Bonanno 6, 56126 Pisa, Italy; simona.sagona@unipi.it (S.S.); a.brogi5@studenti.unipi.it (A.B.); gino.giannaccini@unipi.it (G.G.); 3 Section of Clinical Psychology, Saint Camillus International University of Health and Medical Sciences—UniCamillus, Via sant’Alessandro 8, 00131 Rome, Italy; 4 Department of Biotechnology, Chemistry and Pharmacy, University of Siena, Via Aldo Moro 2, 53100 Siena, Italy; 5 Department of Veterinary Science, Section of Biochemistry, Viale delle Piagge 2, 56124 Pisa, Italy; antonio.felicioli@unipi.it; 6 North-Western Tuscany Region NHS Local Health Unit, Department of Psychiatry, Head Office, Via Cocchi 7/9, 56121 Pisa, Italy

**Keywords:** major depressive episode, DSM-5/SCID-5, psychiatric rating scales, circulating brain-derived neurotrophic factor (BDNF), inflammation indices, blood metabolic parameters

## Abstract

This study seeks to offer a contribution to the method of subtyping major depressed patients by exploring the possible relationships between circulating brain-derived neurotrophic factor (BDNF), different peripheral inflammatory/metabolic markers in the blood and clinical characteristics. Thirty-nine patients, thoroughly diagnosed according to the DSM-5 criteria, underwent a comprehensive set of evaluations encompassing structured interviews, rating scales and a panel of blood tests. Correlation and comparison analyses were carried out by means of non-parametric statistical tests. Concurrently, a principal component analysis was performed to explain biochemical variance. The findings of our research unveiled that leukocyte counts, their ratios and other inflammatory parameters are positively correlated with depression scores. Moreover, we found variations within the BDNF pools of depressed patients. Specifically, higher levels of platelet-poor plasma BDNF (PPP-BDNF) were correlated with augmented inflammatory markers in patients showing specific episode characteristics, whereas reduced platelet BDNF (PLT-BDNF) provided a better indication of the changes that were linked to a diagnosis of long-term depression. Our findings suggest that PPP-BDNF and PLT-BDNF might differentiate depression conditions. They also imply usefulness in appraising peripheral biomarker profiles in patients for a deeper characterization of major depressive episodes. At the same time, it is plausible that they might constitute novel avenues for developing more tailored therapeutic strategies for patients with MDs.

## 1. Introduction

Different depressive episodes can occur across the lifespans of people who are affected by mood disorders (MDs) that represent a common and highly disabling mental condition, and that can be ascribed to a major depressive disorder (MDD) or to bipolar disorders (BDs) [1].

Since the discovery of the first antidepressants (ADs) and the subsequent notion of depression resulting from a “biochemical abnormality”, the most relevant pathophysiological mechanism of MDD was related to a diminished monoamine neurotransmission [2]. Although the monoamine hypothesis of depression is still largely agreed amongst neuroscientists, the pathogenesis of this illness was revised and extended throughout the years, encompassing a multifaceted vision today [3]. Indeed, a body of evidence supports the presence of a dysregulated activation, in different patients and to varying degrees, of neuroendocrine/inflammatory responses during allostatic and homeostatic physiological processes of stress coping [4]. Players of these neuroendocrine loops were found to be disturbed in depression [5], implying that altered biochemical patterns/networks, rather than disturbances of single and separate parameters, might underlie a depressive episode [6]. This could explain the wide heterogeneity of clinical depressive phenotypes [7]. While it is clear that depression is not simply an inflammatory disorder, the imbalance in neuro-immuno-endocrine functions accompanied by an increase in inflammatory biomarkers in depressed individuals is no longer considered just serendipitous. Some investigations revealed that protein complexes defined as “inflammasomes”, intertwined with inflammatory and oxidative stress processes, are linked to depressive symptoms [8]. This hypothesis highlights relevant interplays between behavior and metabolism [9]. For instance, altered behavior can affect the purinergic metabolism that crucially links the production of the high-energy nucleotides ATP and GTP to redox systems and chemical species such as uric acid (UA), which is a byproduct of purine catabolism excreted in urines, but is present in the bloodstream as a powerful antioxidant compound [10]. Within this framework, it is not surprising that somatic conditions such as obesity, diabetes, metabolic or pain syndromes and cardiovascular diseases are frequently associated with depression episodes [5,6,7,8,9,10,11]. Some somatic symptoms can be normalized via a successful AD treatment, which supports the idea that impaired neurotransmission may impact metabolic/redox mechanisms in depression. Concomitantly, dysfunctional arousal states can be linked to inadequate lifestyle and dietary habits, which damage the metabolism, nutritional conditions and antioxidant responses of depressed patients [12]. Recent evidence of changed brain–periphery cross-talks in depression is provided by the significant variations in hematological parameters, such as platelet counts, leukocyte (LK) formulas or their ratios, in MDD [13,14]. Interestingly, at the crossroads between neurotoxicity, inflammation and oxidative stress, there are neuropeptides and neurotrophic factors, particularly the secretory protein brain-derived neurotrophic factor (BDNF) [15]. The BDNF, which is widely synthesized and expressed in the brain, belongs to the neurotrophic family, specifically modulating neuronal plasticity, synapse formation and synapse re-modeling [15]. The BDNF is mostly expressed in the hippocampus and cortex, which are the main brain areas that control cognition, learning, moods and emotions [15]. In addition, it is also expressed in peripheral tissues/organs, particularly in the skeletal muscles, thymus, heart, liver, lung, spleen and vascular smooth muscle cells, as it is produced in blood cells, eosinophils and endothelial cells [16]. Platelets are the main source of circulating BDNF, suggesting the role of this neurotrophin as a mediator of platelet functions and a buffer system during inflammation [16]. Peripheral BDNF indeed exerts tissue-repairing functions and is able to modulate the neuro-immuno-endocrine axis, implying the relevance of investigating it in depression [16]. Most data gathered from central nervous system (CNS) samples reported low brain levels of BDNF in stressed animals and humans, in people who have attempted suicide and in depressed patients [17,18]. Additionally, the administration of ADs augments BDNF brain levels, supporting the pathogenetic role of reduced BDNF in depression [18]. As far as peripheral BDNF is concerned, lower plasma/serum BDNF levels were found in depressed patients, similarly to those present in the CNS, indicating that circulating BDNF may reflect the central neurotrophin counterpart [16]. However, other studies did not confirm these results [16,19], probably because of the intrinsic heterogeneity of the disease and the complexity of the neuroendocrine axes that are potentially involved. Therefore, the appraisal of peripheral BDNF, in the context of determining so-called inflammation/metabolic “signatures” in depression, is still considered important and very challenging. Assessing peripheral BDNF can provide neuropsychiatrists with an additional tool for characterizing/monitoring different clinical presentations, including pharmacological responses. This would enable them to implement more personalized healthcare approaches for patients to prevent symptom relapses and somatic comorbidities, while also considering that at least one-third of patients show a meager or no response to first- and second-generation ADs [20]. Again, all available data further support the idea of depression as a “spectrum disease” defined by inter-individual variations and the existence of symptoms’ “continuum” within patients with MDs and even inside the whole community [7].

According to this effort, the present open, cross-sectional and naturalistic survey aims to assess the possible relationships between clinical features and inflammatory, hematochemical and metabolic parameters, as well as the levels of platelet-poor plasma (PPP) and intra-platelet (PLT) BDNF, within a group of patients with a current major depressive episode (MDE). The possible differences in the biological parameters in the subgroups of patients, based on their episodic or lifetime clinical features, are also appraised. Lastly, to better explain the patients’ biochemical variations, a multivariate analysis is concomitantly conducted.

## 2. Materials and Methods

### 2.1. Subjects

All subjects participating in the study were recruited from both in- or outpatient psychiatric settings where they were treated for an MDE. Thus, all the patients with a current depressive episode were examined for recruitment.

Patients who were enrolled for about one year (January 2019–March 2020) were evaluated by trained psychiatrists through a one-hour-long psychiatric interview in order to assess their detailed clinical presentation with respect to the presence of depressive, manic and anxiety symptoms and lifetime suicidal ideation/attempts.

The MDE occurred in the context of a lifetime diagnosis of MDD, BD of type I (BD-I) or type II (BD-II), in agreement with the criteria of the *Diagnostic and Statistical Manual of Mental Disorders, Fifth Edition* (DSM-5) [1]. The duration of illness and the number of lifetime episodes were appraised from the onset of the disorder until the day of recruitment.

In accordance with the naturalistic design of the study and, in the meantime, to restrain all the possible interfering biases, the adopted inclusion criteria were the following: aged between 18 and 65 years; a history and diagnosis of MDE according to the classification criteria; recruitment at the first visit to our psychiatric clinic (outpatients) or first day of hospitalization (inpatients) prior to any therapeutic intervention, that is, specifically, before any change in current therapies at the time of clinical care and enrollment procedures, considering subjects on stable psychopharmacological therapy over time, with no variation for at least up to one month before the study; having read and accepted the research protocol and signed the study’s informed consent form approved by the Ethics Committee at the University of Pisa. The exclusion criteria were the following: past or present personal history of neurological, rheumatic, infectious, tumor or full-blown metabolic disorders; presence of cognitive alterations and inability to answer the clinical questions or to sign the informed consent form of the study; pregnancy; alcohol abuse or substance abuse in the past 6 months. Patients could choose to leave the study in the case of onset of severe medical illness or clinical signs that were incompatible with the inclusion/exclusion criteria, or withdrawal of informed consent.

### 2.2. Psychiatric Evaluation

The diagnostic and symptomatology assessments were performed using the following tools: the Structured Clinical Interview 5 (SCID-5) [21], which is an interview according to the DSM-5 criteria that is used to recruit patients with MDE, as well as to estimate the episode’s specifiers and severity; the semi-structured interview for MDs (SIMD) [22], for collecting information on demographic data, familiarity, medical history and clinical aspects, such as number and polarity of previous episodes or suicide attempts; the 21-item Hamilton Depression Rating Scale or HAM-D [23]; the Young Mania Rating Scale (YMRS) [24]; the Clinical Global Impression of Severity (CGI-s) Scale [25], which is a global clinical evaluation of the severity of the illness based upon the presence of depressive, counter-polar, anxious and mixed symptoms, and also upon behaviors and functions; the Global Assessment of Functioning (GAF) [26] for the state of patients’ global daily activities.

### 2.3. Determination of Blood Hematological/Biochemical Parameters and Circulating BDNF Levels

#### 2.3.1. Chemicals, Reagents and Instruments

Chemicals and reagents used in the study were of the best analytical grade. Ultra-pure HPLC gradient grade distilled water was employed for all solutions. Optical and fluorescence measurements were carried out using a 96-well plate spectrophotometer (MultiSkan FC Thermo-Scientific, ThermoFisher Scientific, Waltham, MA, USA) and a multimodal Enspire reader (PerkinElmer, Waltham, MA, USA).

#### 2.3.2. Blood Sampling, Plasma and Platelet Separation and Storage Conditions

Peripheral venous blood was collected from each fasting patient between 8.00 and 9.00 a.m. by the nursing staff of the psychiatry unit. Blood withdrawals were carried out while avoiding hemolysis, as part of routine blood tests, according to the local Ethical Committee guidelines for this study. Collected blood was marked and predisposed for the following two different sets of analyses:

(1) About 8 mL/patient was sent to the Laboratory of Clinical Chemical Analysis of the Department of Laboratory Medicine at the University-Hospital of Pisa, Italy, for the determination of hematological/hematochemical parameters.
(2) About 12 mL/patient was sent to the Laboratory of Biochemistry at the Deartment of Pharmacy, University of Pisa, to determine PPP-BDNF and PLT-BDNF.

The routine blood examinations included the following parameters: LK, LK-subset and platelet counts, mean platelet volume (MPV), C-reactive protein (CRP), erythrocyte sedimentation rate (ESR,) UA, total bilirubin, blood glucose and lipoproteins. Platelet-to-lymphocyte ratio (PLR), neutrophil-to-lymphocyte ratio (NLR) and monocyte-to-lymphocyte ratio (MLR) were directly calculated from blood cell counts. Some UA determinations were carried out at the Department of Pharmacy (biochemistry laboratory) by means of a fluorometric assay kit purchased from Cayman Chemicals (Ann Arbor, MI, USA).

For BDNF measurements, EDTA blood samples were treated, for no more than 30 min after collection, to separate PPP from pellets containing whole platelets, as previously indicated [27]. This was carried out to avoid artifacts linked to abnormal BDNF release after blood collection and during sample separation [28]. PPP and whole platelets were aliquoted and separately stored at −80 °C until assay.

#### 2.3.3. Preparation of Platelet Soluble Fractions

On the day of the BDNF assay, whole platelet pellets were thawed and homogenized to obtain platelets’ soluble fractions; platelets were immediately placed on ice and homogenized in 6 mL ice-cold lysis buffer containing protease inhibitors (1:500, *v:v*) by means of an ultrasound mechanical device, and centrifuged as previously described [27]. The resulting supernatant was used for the PLT-BDNF assay.

#### 2.3.4. BDNF Sandwich ELISA Assay

A sandwich ELISA kit, developed for the determination of the free mature form of BDNF, was employed (Biosensis, Mature BDNF Rapid TM, Thebarton, Australia). On the day of assay, both a per-patient aliquot of thawed PPP and a per-patient aliquot of freshly prepared platelet soluble fraction were properly diluted in the assay buffer, according to the kit guidelines. After incubation at RT and repeated washing steps, BDNF was revealed by adding a biotinylated secondary anti-BDNF antibody and a horseradish peroxidase enzyme (HRP) bound to a streptavidin complex. The revelation reaction was started by the addition of tetramethylbenzidine (TMB), an HRP substrate, and halted via a concentrated strong acid. Absorbance was read at 450 nm using the MultiSkan spectrophotometer.

For interpolating BDNF unknowns, a calibration curve was built using a 4 PL non-linear regression equation, obtaining PPP-BDNF and PLT-BDNF concentrations as ng/mL, after correction for the dilution factor.

PLT-BDNF contents were then normalized for the total protein amount (mg/mL) present in each final platelet soluble fraction and converted into ng/mg proteins. Protein amount was measured using the Bradford method [29], using γ-globulins as the standard.

Normalization of PLT-BDNF (ng/mL) for protein content in platelet soluble fractions was carried out to restrain the impact on the results of both the interindividual variability of platelet counts and the recovery of the extraction procedure implemented.

#### 2.3.5. Statistics and Calculations

Demographic, clinical and biological data were presented as the mean ± standard deviation (SD), medians and ranges (min and max values). Descriptive, inferential statistics and calibration curves for each quantitative assay conducted in the study were all performed using the GraphPad Prism^®^ Software (Version 7.0.2, San Diego, CA, USA).

Given the expected variability of the data due to the intrinsic heterogeneity of the illness presentations, and considering that the homoscedasticity of variances as well as the normality of the distributions were not respected for many of the variables examined, non-parametric inferential statistics were chosen for elaborating all collected data; Spearman’s correlations were performed to estimate the relationships between the patients’ biochemical variables and psychometric scores, while Mann–Whitney and Kruskal–Wallis tests were used for comparison tests. For the same reasons, to avoid type-II errors, a two-tailed statistical significance was preset at *p* ≤ 0.05, whereas *p*-values within the interval 0.05 < *p* ≤ 0.1 were considered trends to significant results. For a better interpretation of biological variations, a multivariate principal component analysis (PCA) on correlations, involving the hematological/biochemical dataset, was also carried out with the collaboration of the Laboratory of Biochemistry of the Department of Veterinary Sciences, University of Pisa, by means of the Statistical Software JMP (Version 7.0, SAS Institute Inc., Cary, NC, USA).

## 3. Results

### 3.1. Subjects

Thirty-nine subjects of both the male and female sexes (27 women and 12 men) were found to meet the adopted recruitment criteria and participated in the study. As shown in Table 1, the enrolled patients displayed a female-to-male ratio of about 2:1. Furthermore, they were middle aged (mean age = 52 years), moderately overweight (mean body mass index, BMI = 27 Kg m^−2^), predominantly married or living with a partner (66%), unemployed (74%), and had middle school education (11 years on average). Their diagnosis was received about twenty years earlier, at a mean age of 30 years. Moreover, no patient was at his/her first episode at the time of the study, with a history of eight lifetime episodes on average, indicating the preponderance of a chronic MD as defined by recurrent episodes (Table 1).

The prevalent lifetime diagnoses were BD of type II (BD-II), followed by MDD and BD of type I (BD-I). In particular, 31 of the 39 patients or 79.5% (21 women and 10 men) were suffering from a BD, while 8 of them or 20.5% (6 women and 2 men) had a diagnosis of MDD; 7 of the 31 patients suffering from a BD were diagnosed with BD-I (17.95%, 5 men and 2 women) whereas 24 of them with BD-II (61.54%, 5 men and 19 women). The majority of women were suffering from MDD and BD-II, while men were prevailing amongst BD-I subjects. Familiarity of MDs was observed in almost all subjects (34, 87.2%; 24 women and 10 men), and familiarity of depression was found in 28 of the 34 subjects (71.8%; 20 women and 8 men); familiarity of mania was observed in only 6 subjects (15.4%; 4 women and 2 men). Furthermore, they had a prevalent familiarity of depression, with a first episode polarity mainly of the depressive type, implying their high vulnerability to a depressed mood. Most of the patients (92%) had been hospitalized during periods of illness, while 31% of them had attempted suicide, especially women, with 15.3% of them having attempted suicide more than once. Among those who attempted suicide, none of them had attempted suicide during the current depressive episode, with the last attempt carried out at least a year before the present investigation. Those who attempted suicide had a significantly lower number of episodes than non-attempters.

Table 2 displays the current episode characteristics of the enrolled patients; “Current Severity” according to the DSM-5 criteria enabled us to identify a severe episode in 59% of patients and a moderate one in 41%, with none presenting mild episode features. Moreover, according to the SCID-5 interviews, we identified patients currently suffering from a depressive episode with the prevalent specifier “anxious distress” (79.4%). Indeed, the most frequent psychiatric comorbidity was panic disorder (64%), followed by generalized anxiety disorder (10%) and obsessive compulsive disorder (7%).

### 3.2. Psychiatric Rating Scales and Symptom Presentation

All recruited patients showed depressive symptoms with scores of ≥17 (mean + SD: 25 ± 5) on the HAM-D scale, and a mild manic component (Table 3). The results were classified as severe, as assessed by the CGI-s scale score (mean ± SD: 5.0 ± 0.6), and as moderate-to-severe functional impairment (GAF mean score ± SD: 45 ± 8); the total YMRS score (mean ± SD) was 3.0 ± 3.0.

### 3.3. Inflammatory, Hematological–Metabolic Indexes and BDNF Values

Table 4 reports the results obtained at the Clinical Chemistry Laboratory, with their fasted physiological values, as well as the PPP-BDNF and PLT-BDNF values gathered at the Laboratory of Biochemistry. The clinical–chemical parameters were measured in 29 out of the 39 patients recruited. As far as the inflammatory, hematologic and metabolic results are concerned, it is worth noting that the patients had mean CRP values that were higher than the normal threshold. In detail, 18 of the 29 examined patients (65.5%) had CRP values of ≥0.1 mg/100 mL. According to a previous work [30], the following three different groups of patients could be distinguished on the basis of the amount of this inflammatory parameter: group 1 (n = 10, 34%), mainly composed of women, displayed normal CRP levels (CRP < 0.1 mg/100 mL); group 2 (n = 13, 45%), mainly composed of men, showed slightly increased values (0.1 ≤ CRP ≤ 0.5 mg/100 mL), whereas group 3 (n = 6, 21%), overall women, showed strongly increased amounts (CRP > 0.5 mg/100 mL). A Kruskal–Wallis test, followed by Dunn’s post hoc comparison, showed that group 3 had strongly increased CRP values (mean ± SD = 3.72 ± 4.5) than group 1 (mean ± SD = 0.023 ± 0.028, *p* < 0.0001); group 2 had also greater CRP values (mean ± SD = 0.204 ± 0.087) than group 1 (*p* < 0.01).

As for the patients’ metabolic profiles, the glucose, UA, bilirubin, triglyceride and total cholesterol were within the normative values. However, at least 40% of patients had HDL levels < 65 mg/100 mL and LDL values > 115 mg/100 mL. The hematological profiles showed that the white blood cells (WBC) were overall within the physiological ranges, but the leukocyte counts were beyond normal ranges in some patients, being lower in 14% of the total patients and higher in 17% of them. Similar results were obtained for the neutrophil counts. Also, in a few patients, the NLR was found altered with respect to the physiological thresholds, and the PLR resulted higher than the normal ranges.

As regards the neurotrophin circulating levels measured in the patients, the PPP-BDNF showed a high variability. Prior to the standardization for the protein content in platelet soluble fractions, the PLT-BDNF (mean ± SD) was 13.34 ± 6.65 ng/mL, which is much higher in comparison to PPP-BDNF, its extracellular counterpart, showing, on average, a ratio between the platelet and plasma components with a mean value of 15 (range: 1.2–99) [30].

### 3.4. Correlations and Comparison Analyses between Clinical Demographic Results and Biological Parameters

**Figure 1:**
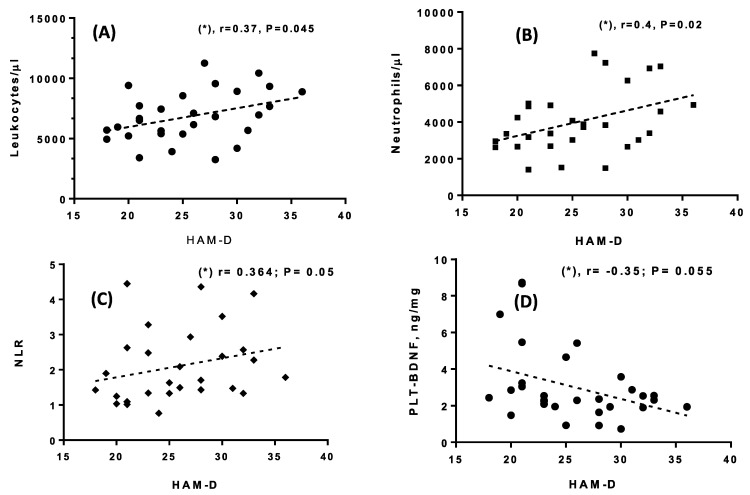
(**A**–**D**) Spearman correlations between blood cell parameters and total HAM-D scores (n = 29). (*): *p* ≤ 0.05. Dotted lines are graphical representations of the correlation trends, obtained from the best linear fit.

No significant correlation or comparison was found for variables such as age, gender, illness duration or number of lifetime episodes. As well, no substantial correlation was obtained between the BMI and clinical scales, nor between the BMI and demographic characteristics of the patients. Only a significant correlation between the clinical demographic results was found, showing that the YMRS scores were positively correlated with the number of lifetime episodes experienced (r = 0.5, *p* = 0.001). Correlations between the patients’ biochemical results and psychometric scores revealed significant and positive relationships between the LK, neutrophils, the ratio NLR and the HAM-D total score, as depicted in Figure 1A–C. The YMRS only tended to be positively correlated with the PLR (*p* = 0.08). Moreover, a negative correlation was found between the PLT-BDNF and the HAM-D score (Figure 1D). No other biological parameter was found to be associated with clinical features. Despite the PPP-BDNF levels being significantly and positively correlated with the PLT-BDNF (r = 0.37, *p* < 0.05), this circulating neurotrophin component only showed a non-significant trend for lower values with respect to higher HAM-D scores (r = −0.26, *p* > 0.1).

Concerning the correlations between the anthropometric/demographic data and biochemical results, only the BMI was found to be positively correlated with the CRP (r = 0.377; *p* = 0.04).

**Figure 2:**
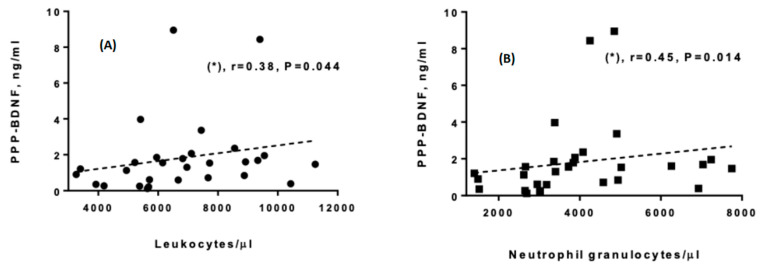
(**A**,**B**). Significant Spearman correlations between PPP-BDNF and blood cell parameters (n = 29), (*): *p* < 0.05. Dotted lines are graphical representations of the correlation trends, obtained from the best linear fit.

Spearman correlations were also carried out between the different biochemical parameters investigated. The PPP-BDNF was positively and significantly correlated with the LK (r = 0.38, *p* = 0.044) and neutrophil counts (r = 0.45, *p* = 0.014) (Figure 2A,B), although it was not correlated with any assessment scales.

A trend towards significant positive correlations was also found between PPP-BDNF and NLR, PLR or platelet number.

**Figure 3:**
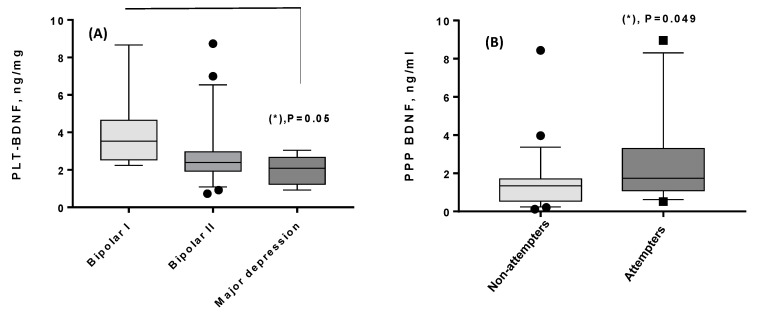
(**A**,**B**) Significant comparisons obtained for the two BDNF components and lifetime clinical features. (**A**) PLT-BDNF (ng/mg protein, mean ± SD, median, range) was 4.1 ± 2.16, 3.53, 2.24–8.67 in 7 BD-I patients; 2.96 ± 1.97, 2.4, 0.73–8.73 in 22 BD-II patients; 1.98 ± 0.8, 2.1, 0.93–3.0 in 5 MDD patients. (*): Kruskal–Wallis analysis and post hoc Dunn’s test: lower PLT-BDNF in MDD vs. BD-I, *p* ≤ 0.05. (**B**) PPP-BDNF (ng/mL, mean ± SD, median, range) was 1.55 ± 1.68, 1.34, 0.12–8.43 in 27 non-attempter patients and 2.76 ± 2.6, 1.74, 0.52–8.95 in 12 patients who have attempted suicide. (*): Mann–Whitney U-test: higher PPP-BDNF values in patients who have attempted suicide vs. those who have not, *p* < 0.05.

In addition to correlations, the biological results were compared on the basis of lifetime illness characteristics (Table 1), as well as in respect to the episode specifier “Current Severity” (Table 2). The following significant results were obtained for lifetime features: lower PLT-BDNF amounts were found in patients with a diagnosis of MDD than in those with BD-I Figure 3A), while higher PPP-BDNF levels were obtained in patients who have attempted suicide in comparison with patients who have not (Figure 3B).

**Figure 4:**
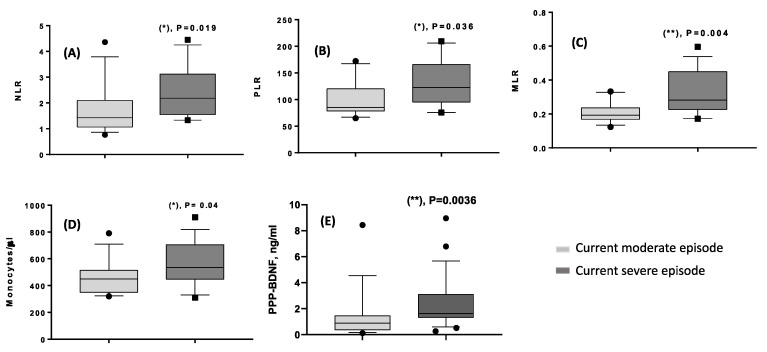
(**A**–**E**) Significant comparisons obtained in 23 patients with current moderate episodes vs. 16 severe depressive episodes, Mann-Whitney U-test (*): *p* < 0.05; (**): *p* < 0.01. (**A**) NLR (mean ± SD, median, range) was 1.72 ± 1.0, 1.425, 0.764–4.36 in moderate episodes; 2.39 ± 0.99, 2.18, 1.33–4.45 in severe episodes. (**B**) MLR (mean ± SD, median, range) measured 0.21 ± 0.062, 0.193, 0.124–0.33 in moderate episodes; 0.34 ± 0.135, 0.282, 0.172–0.596 in severe episodes. (**C**) PLR (mean ± SD, median, range) was 101.6 ± 33.8, 85.2, 64.9–172.3 in moderate episodes; 133 ± 42.7, 123, 75.7–209.5 in severe episodes. (**D**) Monocyte count (cells/μL, mean ± SD, median, range) was 449.2 ± 133, 450, 320–790 in moderate episodes; 570 ± 162, 535, 310–910 in severe episodes. (**E**) PPP-BDNF (ng/mL, mean ± SD, median, range) was 1.35 ± 2.0, 0,89, 0.12–8.43 in moderate episodes; 2.31 ± 2.0, 1.62, 0.275–8.95 in severe episodes.

Comparisons based upon the current episode severity revealed several significant results. Increased values of NLR, PLR and monocytes were reported in patients with severe vs. moderate episodes, as depicted in Figure 4A–C; in addition, even higher values of MLR and PPP-BDNF were found in patients with a severe episode than those with a moderate one (Figure 4D,E).

### 3.5. Principal Component Analysis (PCA) of Biological Results

The variables that are the most informative of the total biological data variance were investigated by means of the multivariate PCA model on correlations, which enabled us to restrict the impact of possible collinearity among the various biochemical panels on the results. No relevant outlier was found by the “Mahalanobis Distances” test.

**Figure 5:**
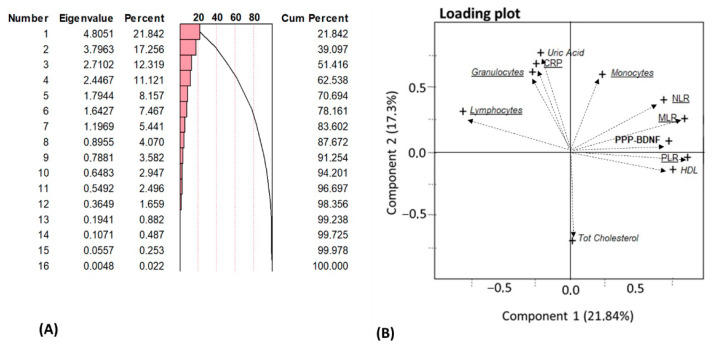
(**A**,**B**) Principal component analysis (PCA) and variance of the biological dataset. (**A**) Eigenvalue results with the percent of variance explained by the obtained principal components; the first seven components (eigenvalues > 1) explained 83.6% of the total variance. PCA1 and PCA2 explained 39% of the total variance. (**B**) 2-D loading plot graph with eigenvectors, loading values and the corresponding biological variables examined; the figure depicts metabolic parameters in italic font, inflammatory parameters in underlined capital letters, hematological parameters in underlined italic font and neurotrophic parameters in bold letters. For the sake of clarity, the figure shows only the parameters with loading scores higher than 0.5 and lower than −0.5; for the same reason, LK was also omitted from the graph—this parameter’s resulting plots were almost superimposed to the granulocytes.

About 84% of the result variability concerning the biological assessments was due to the first seven principal components, when considering components with eigenvalues of >1 (Figure 5A).

Figure 5B depicts the loading plot results with eigenvectors associated with the first two components, displaying moderate-to-strong loading scores (−0.8 ≤ x < 0.8); the MLR, PLR, NLR ratios, HDL and PPP-BDNF values were found to be positively linked, while the lymphocyte count was negatively correlated with the PCA1 (21.84% of global data variance). The PCA2 (17.26% of global data variance) was instead positively featured by the UA, CRP and the LK/granulocyte count, and was negatively featured by the total cholesterol.

Table 5 reports the variables that were significantly correlated with the first two components, which accounted for ≈39% of the total data variance, or the PLR, NLR, MLR, PPP-BDNF, lymphocytes, LK/neutrophils, UA, CRP and cholesterol profiles.

## 4. Discussion

The results of the present research led to a series of intriguing findings. First, the clinical–chemical blood tests, while revealing that the patients had CRP values higher than normal thresholds, support the notion that a mild inflammation can underlie an MDE, which is probably linked to inflammasome activation [8]. This is in line with the observation that depressive/anxious symptoms might be linked to mild increases in the CRP levels [31,32]. However, the CRP was positively and significantly correlated with the subjects’ BMI, as already observed in the anxious depressed patients [32], but not with the episode’s severity or clinical scale scores, which is in apparent contrast with a previous work [33], suggesting that the increase in CRP values that was found in 65.5% of the patients was mainly related to their depressive condition rather than to the symptom score, as assessed using the rating scales.

Conversely, the WBC counts or inflammatory indices such as the NLR were found to be positively and directly related to depressive/manic symptoms, while also being increased during a current severe episode in our sample. These are important findings that are in agreement with other studies where these same parameters were related to the presence of somatic comorbidity in depression, being more elevated with cardiovascular risk [34], severity of episodes [34,35] and suicide attempts [36]. Our LK, NLR, MLR and PLR results are also in line with the proposal of these indices as economic and promising tools for monitoring MDs and MDEs [14,37]. Again, the observed increase in the NLR, MLR and PLR in severe episodes might specifically have a prognostic relevance for MDs. Interestingly, some other previous studies have found low LK counts in patients at their first depression diagnosis, suggesting that MDs can be defined according to the LK population profiles dependent on clinical states and related inflammatory paths [38].

To our knowledge, the present work is the first that has simultaneously evaluated both the extracellular (PPP) and intra-platelet (PLT) components of the free mature BDNF in patients suffering from MDs with symptom relapse and a current MDE. Most previous studies have assessed the serum and/or plasma BDNF pools, reporting lower neurotrophin in patients [16]. Conversely, a few investigations were performed inside platelets, mainly reporting reduced BDNF in depressed patients [16], a finding that is congruent with works conducted on serum BDNF, which is considered to be dependent on platelet activation, being the amount released from α-granules [16]. Plasma BDNF levels, which are much lower than the serum ones, depend instead on the “basal” release from platelets, but also from LK, vessel-endothelial cells and from the brain, too. On the other hand, it should be also mentioned that intra-platelet contents and serum levels do not always correspond to each other. Indeed, the PLT-BDNF measured in soluble fractions can result not only from the above-reported extracellular sources, but even from non-released platelet reserves also deriving from megakaryocyte production [16,39,40]. Our PPP-BDNF values were in agreement with these previous works, proving to be highly variable [16,28,30]; in addition, prior to normalization, the PLT-BDNF amounts were found to be variably higher than the extracellular component, being up to almost 100-fold higher, which is a result that is also in line with the current literature [16,28]. Additionally, the present BDNF results suggest that the trajectories of plasma and intra-platelet amounts may provide different indications to clinicians. Indeed, the PLT-BDNF was found to be inversely correlated with the HAM-D scores, that is to say, it decreases in patients with the most severe depressive symptoms during an MDE. The PLT-BDNF did not correlate with the inflammatory/metabolic markers investigated herein, instead resulting in lower levels in MDD patients than in BDI patients. Therefore, it can be hypothesized with caution that the PLT-BDNF pool could constitute a potential biomarker of conditions that are more directly related to depression and an MDD diagnosis. Conversely, PPP-BDNF did not correlate with any psychometric data, but it was found to be positively correlated with the LK and neutrophil granulocytes, two parameters that were positively correlated with the HAM-D scores and considered inflammatory indices in MDs [14,34,37,41]. Additionally, PPP-BDNF was increased in the patients who have attempted suicide during their lifetime, as well as in patients with a current severe episode. Our results indicate that the increase in the PPP-BDNF concentration would be secondary to the activation of specific inflammatory paths in subsets of affective patients showing peculiar episodic clinical conditions and/or mental/somatic vulnerabilities. Within this framework, it should also be pointed out that even higher plasma BDNF levels were previously observed in MDs and related syndromes such as in depressed patients [19], in BDNF Met carriers with chronic depression [42], in patients with a mixed-state episode compared with patients with a depressive episode [43], in treatment-resistant BD patients displaying high interleukin-1β [44], in pain perception disturbances [45] and in adjustment disorders [46]. At the same time, it also cannot be ruled out that in the context of the overall low BDNF reserves commonly reported in depression [47,48,49], positive correlations between PPP-BDNF and pro-inflammatory indices might derive from a condition of allostasis overload in subgroups of MD patients displaying altered/blunted counter-responses during inflammation, such as the release of BDNF. Furthermore, it should be mentioned that our patients differed from those examined in other reports, as they had a prevalent diagnosis of BDs with chronic features and a current anxious/depressive episode that might be due to distinct neurobiological substrates [50].

Overall, the present results imply that important aspects of the biology of BDNF should be further investigated, as suggested by some authors [49,51], for a deeper understanding of its role in the pathogenesis of depressive episodes, such as the genetic, transcriptional, epigenetic and post-translational variance of this neurotrophin. They also highlight new insights for the possible therapeutic application of molecules targeting BDNF and its regulation [52] in MDs.

The results from the multivariate PCA analysis provide additional information on biological data by restraining variable redundancy and by limiting the possible collinearity between some of the variables investigated. The first two principal components explained a relatively low percentage of total variance, but the 2D loading plot values had moderate-to-strong results for variables such as the WBC and platelet ratios, PPP-BDNF, UA, CRP, HDL and total cholesterol. Through PCA, it can be hypothesized that the first component identifies a subgroup of patients presenting higher WBC ratios and PPP-BDNF together with lower lymphocyte counts and normal HDL values. The second component suggests the presence of a subgroup of patients showing higher UA, CRP and LK/granulocytes and monocytes with normal total cholesterol values. Therefore, the PCA supported the variation of the CRP, inflammatory and metabolic factors within the patients suffering from a depressive episode. Moreover, this analysis showed again that PPP-BDNF is the neurotrophin component most involved in variations that are related to inflammatory patterns; as well, PCA showed that metabolic indices of inflammation, like higher UA and CRP, can be found independently of altered lipoprotein profiles, while being in association with increased LK formulas.

This open and cross-sectional study presents some limitations that should be acknowledged. The main bias is that it was carried out in a small sample of patients with MDE; however, the selection criteria were quite strict and, according to us, permitted us to recruit a group of patients that adequately reflects the clinical reality. Again, although all patients were assessed before any pharmacological intervention, given the scarce numerosity, we could not analyze the impact of past psychotropic drugs on biomarkers. Another important limitation is that no data on smoking behavior or physical activity were recorded.

In summary, the present findings, despite their inherent limitations, support the usefulness of implementing the easily achievable measure of blood cell counts and metabolic inflammation indices, together with that of circulating BDNF, with the aim of optimizing the clinical care and follow-up of patients with MDs. Such parameters might be of help to monitor the episodes’ severity, the presence of activated inflammatory paths and possibly even the vulnerability towards specific somatic diseases. In addition, they seem to suggest novel avenues of future interventions in MDs.

## Figures and Tables

**Table 1:** Main demographic, anthropometric and illness characteristics of the total sample of patients (n = 39) according to sex (12 men and 27 women).

Patients	Total, Mean ± SD (Median; Range)	Men, Mean ± SD (Median; Range)	Women, Mean ± SD (Median; Range)
Age (years)	52 ± 14 (55; 18–65)	53 ± 15 (60; 23–65)	52 ± 13 (53; 18–65)
BMI (kg/m^2^)	27 ± 5 (26; 19–37)	28 ± 6 (28; 20–37)	27 ± 5 (26; 19–37)
Age at onset (years)	29 ± 12 (29; 6–61)	29 ± 14 (23; 12–56)	30 ± 12 (29; 6–61)
Illness duration (years)	24 ± 12 (25; 2–52)	26 ± 14 (24; 6–52)	22 ± 11 (25; 2–45)
Number of lifetime episodes	8 ± 5 (6; 1–21)	11 ± 6 (10; 4–20)	7 ± 5 (6; 1–21)

**Table 2:** Characteristics of the current depressive episode.

Patients	Total, n (%)	Men, n	Women, n
Current Episode Severity (DSM-5) *			
Severe (8–9 DSM-5 criteria satisfied)	23 (59)	7	16
Moderate (6–7 DSM-5 criteria satisfied)	16 (41)	5	11
SCID-5 Specifier “with anxious distress”			
Yes	31 (79.5)	8	23
No	8 (20.5)	4	4
SCID-5 Specifier “with mixed features”			
Yes	14 (36)	2	12
No	25 (64)	10	15
SCID-5 Specifier “with melancholic features”			
Yes	13 (33)	4	9
No	26 (67)	8	18
SCID-5 Specifier “with psychotic features”			
Yes	6 (15.4)	2	4
No	33 (84.6)	10	23
SCID-5 Specifier “with atypical features”			
Yes	4 (10.3)	0	4
No	35 (89.7)	12	23
SCID-5 Specifier “with seasonal pattern”			
Yes	3 (7.7)	2	1
No	36 (92.3)	10	26

* Following the DSM-5 indications, the designation of “mild”, “moderate”, or “severe” episode was assigned not only on the basis of the number of criteria satisfied, but also on the basis of the impact of the symptoms on the patient’s suffering and overall function.

**Table 3:** Psychopathological assessment and rating scale scores of the current episode.

Patients	HAM-D	YMRS	CGI-s	GAF
Total	25 ± 5 (26; 17–36)	3.0 ± 3.0 (2; 0–12)	5.0 ± 0.6 (5; 4–6)	45 ± 8 (45; 30–60)
Men	26 ± 4 (25.5; 20–32)	3.0 ± 3.0 (3; 0–9)	4.9 ± 0.5 (5; 4–6)	48 ± 5 (47; 41–58)
Women	25 ± 5 (26; 17–36)	3.0 ± 3.0 (2; 0–12)	5.0 ± 0.6 (5; 4–6)	44 ± 9 (43; 30–60)

Data are presented as mean ± standard deviation (SD), median and range (in parentheses).

**Table 4:** Inflammatory/metabolic/hematologic indices and circulating BDNF levels (mean ± SD, median; range) in the total sample of men and women.

	Total	Men	Women	Normal Levels
CRP mg/100 mL	0.9 ± 2.45 (0.15; <0.01–13)	0.442 ± 0.67 (0.17; <0.01–1.93)	1.23 ± 3.19 (0.1; <0.01–13)	≤0.5
ESV mm/h	15 ± 17 (8.5; 2–76)	12.9 ± 12.68 (6; 2–37)	16.1 ± 19.5 (10.5; 2–76)	<25
T-cholesterol mg/100 mL	178 ± 39 (179; 65–225)	168.9 ± 44.9 (174.5; 65–225)	183.8 ± 33.8 (182; 96–224)	<200
LDL mg/100 mL	116 ± 33 (119; 34–180)	115.8 ± 45.21 (118; 34–180)	116.6 ± 22.8 (119; 57–144)	<115
HDL mg/100 mL	49 ± 17 (49; 24–100)	40.75 ± 12.6 (43.5; 24–67)	55.53 ± 17.7 (54; 25–100)	>65
TG mg/100 mL	129 ± 53 (136; 46–280)	139 ± 69 (142; 52–280)	122 ± 38 (129; 46–186)	10–150
Glucose mg/100 mL	90 ± 23 (82; 67–163)	95.2 ± 28.4 (86.5; 70–163)	86.1 ± 18.72 (82; 67–146)	<125
Uric acid mmol/L	0.29 ± 0.09 (0.28; 0.16–0.47)	0.32 ± 0.08 (0.28; 0.2–0.47)	0.27 ± 0.09 (0.24; 0.16–0.43)	0.14–0.34
Total bilirubin mg/100 mL	0.5 ± 0.23 (0.44; 0.11–0.97)	0.57 ± 0.22 (0.51; 0.28–0.97)	0.44 ± 0.22 (0.42; 0.11–0.97)	≤1.2
PLT cells^10^3^/μL	229 ± 65 (223; 120–389)	204.3 ± 47.1 (198.5; 120–310)	246 ± 72.13 (244; 123–389)	140–450
MPV	11 ± 0.9 (10.75; 9.4–12.8)	11.1 ± 0.93 (11.1; 9.4–12.7)	10.8 ± 0.9 (10.6; 9.5–12.8)	8.0–11.9
LK cells/μL	6830 ± 2086 (6670; 3260–11,250)	7238 ± 2191 (6815; 4190–11,250)	6542 ± 2025 (6510; 3260–9550)	4000–11,000
Neutrophils cells/μL	4022 ± 1719 (3720; 1400–7750)	4338 ± 1745 (3560; 2650–7750)	3799 ± 1718 (3840; 1400–7240)	1800–7000
Eosinophils cells/μL	190 ± 119 (170; 0–440)	198.4 ± 126.2 (165; 0–440)	183.5 ± 117.8 (170; 10–410)	≤700
Basophils cells/μL	23 ± 16 (20; 0–80)	15.8 ± 9 (20; 0–30)	28.8 ± 18.7 (20; 10–80)	≤200
Lymphocytes cells/μL	2060 ± 676 (1990; 1030–4100)	2121 ± 664.5 (2020; 1030–3061)	2017 ± 701 (1990; 1040–4100)	900–4500
Monocytes cells/μL	516 ± 159 (500; 310–910)	519.2 ± 146.8 (515; 320–780)	513.5 ± 172 (500; 310–910)	100–1200
NLR	2.09 ± 1.0 (1.71; 0.76–4.45)	2.17 ± 0.84 (2.24; 1.093–3.52)	2.03 ± 1.17 (1.5; 0.76–4.45)	0.78–3.5
MLR	0.275 ± 0.12 (0.23; 0.12–0.6)	0.27 ± 0.125 (0.25; 0.12–0.51)	0.28 ± 0.13 (0.23; 0.17–0.6)	0.1–0.59
PLR	119 ± 41 (113.5; 65–209)	105 ± 40 (83.14; 64.9–174.2)	128.7 ± 40.7 (118; 70–209)	40–200
PLT-BDNF ng/mg proteins	3.05 ± 1.95 (2.44; 0.73–8.73)	3.2 ± 2.2 (2.54; 0.73–8.67)	2.97 ± 1.83 (2.4; 0.92–8.73)	-
PPP-BDNF ng/mL	2.075 ± 2.22 (1.54; 0.12–8.95)	1.41 ± 1.04 (1.5; 0.25–3.97)	2.37 ± 2.53 (1.6; 0.12–8.95)	-

From CRP to PLR: total = 29; PLT-BDNF and PPP-BDNF: total = 39.

**Table 5:** Multivariate principal component analysis (PCA) on correlations—biological results.

Variables	E 1	E 2
PLT	0.093	0.036
VES	0.013	0.144
CRP	−0.108	*0.359*
Tot Chol	0.001	*−0.354*
HDL	*0.304*	−0.081
LDL	−0.063	−0.258
Triglycerides	−0.240	−0.027
Glucose	−0.209	−0.070
UA	−0.082	*0.381*
Leukocytes	−0.112	*0.354*
Neutrophils	−0.021	*0.333*
Basophils	0.039	−0.010
Eosinophils	−0.119	0.218
Lymphocytes	*−0.358*	0.169
Monocytes	0.144	*0.319*
MPV	0.011	0.008
NLR	*0.301*	0.209
PLR	*0.391*	−0.067
MLR	*0.399*	0.128
Total Bilirubin	−0.122	−0.097
PPP-BDNF	*0.344*	0.053
PLT-BDNF	0.243	0.090

E 1,2 = Eigenvectors; italic and underlined values are those with the strongest loading (correlations with the first two principal components, explaining about 39% of data variance).

## Data Availability

The data presented in this study are available in article or can be requested from the corresponding authors.
